# A Retrospective Analysis of Cyanoacrylate Injection versus Hemoclip Placement for Bleeding Dieulafoy's Lesion in Duodenum

**DOI:** 10.1155/2018/3208690

**Published:** 2018-03-26

**Authors:** Yu Jiang, Julong Hu, Ping Li, Wen Jiang, Wenyan Liang, Hongshan Wei

**Affiliations:** ^1^Department of Gastroenterology, Beijing Ditan Hospital, Capital Medical University, No. 8 Jingshun East St., Chaoyang District, Beijing 100015, China; ^2^The Endoscopy Center, Huangshan Shoukang Hospital, Meilin Rd. Economic & Technological Development Zone, Huangshan, Anhui Province 245000, China; ^3^Department of Gastroenterology, Huairou Hospital, Qingchun Rd., Huairou, Beijing 101400, China

## Abstract

**Background:**

Duodenal Dieulafoy's lesion (DL) is a rare disease that may lead to lethal hemorrhage in the upper gastrointestinal tract. The best technique for endoscopic intervention still remains unclear. In the present study, we performed a retrospective analysis of cyanoacrylate injection versus hemoclip placement for treating bleeding DLs.

**Materials and Methods:**

We retrospectively analyzed eighteen patients from three medical centers between October 2008 and February 2016; six patients received cyanoacrylate injection, while hemoclips were placed in 12 patients during the upper gastrointestinal endoscopy.

**Results:**

All patients received first endoscopic examination and/or endotherapy within 12 hours of admission to hospital. No difference was observed in the primary hemostasis rate or the recurrent hemorrhage rate between the cyanoacrylate injection (CI) group and the hemoclip placement (HP) group, except that in one patient from the HP group melena was found three days after the first endotherapy. This patient received cyanoacrylate injection once again.

**Conclusion:**

Both cyanoacrylate injection and hemoclip placement are effective in treating duodenal DL, and neither of them causes significant side effects.

## 1. Introduction

First described in 1898 by Dieulafoy, DL was identified in two patients with fatal upper gastrointestinal hemorrhage without ulceration. The incidence of this disease is rare; nonetheless, it can result in serious gastrointestinal bleeding [1]. In most patients, it is presented as melena, hematemesis, and hematochezia, and it accounts for 1–5.8% cases of acute upper GI bleeding [2]. With the advances in endoscopic techniques, the mortality of DL patients has been reduced from 80% to 8%, and surgical intervention is only considered in cases of failed endotherapy [3].

At the beginning of this century, the first perspective randomized-controlled study demonstrated that hemoclip and band ligation are equally effective compared to injection therapy for DL bleeding [4]. The overall hemostasis rate in hemoclip therapy has been shown to be up to 95% [5], but the ratio of emergent surgery is still above 5%, according to a recent multicenter report [6]. Furthermore, currently, there are no cohort studies on the effectiveness of cyanoacrylate injection for the treatment of duodenal DLs, since most of DLs are located in a proximal stomach. The best technique for endoscopic intervention in DL is still not clear [3]. In the present study, we performed a retrospective analysis of cyanoacrylate injection versus hemoclip placement for treating bleeding DLs in the duodenum.

## 2. Patients and Methods

### 2.1. Study Design

The present study was a retrospective cohort analysis. The study protocol was approved by the Ethics Board of Beijing Ditan Hospital, Capital Medical University. Between October 2008 and February 2016, a total of 18 DL patients from three medical centers were enrolled in the study. All the patients received endoscopy within 12 hours of hospitalization and were diagnosed with duodenal DLs. The last follow-up was 3 months after the endoscopy treatment.

### 2.2. Patients

The diagnosis of duodenal DL was based on recently reported criteria [7, 8] including (i) active arterial spurting or bleeding from minute defect (<3 mm); (ii) a protruding vessel within nearly normal mucosa; and (iii) a fresh, densely adherent clot within the normal-appearing mucosa. The patients' age, sex, blood pressure, hemoglobin, prothrombin time (PT), concurrent disease, location, the type of bleeding stigmata, and final outcomes were analyzed. Primary outcomes, including the primary hemostasis rate and rebleeding rate, were compared between the two groups. The secondary outcomes such as the number of endoscopic sessions, need for emergent surgery or transcatheter arterial embolization, bleeding-related deaths, transfusion requirements, hospitalization period, and survival time information were also retrospectively compared between the two groups.

### 2.3. Endoscopic Therapy

Data were collected according to following criteria: (i) endoscopic treatments were performed by gastroenterologists with at least 3 years of endoscopic experience; (ii) patients received endoscopic examination within 12 hours of hospitalization; (iii) duodenal DL diagnosis was confirmed by endoscopy; (iv) after being diagnosed, the patient received either cyanoacrylate injection (CI group) or hemoclip placement (HP group); and (v) 3–10 days after the endoscopic treatment, patients received the first endoscopic follow-up, and after 1–3 months, they received the second endoscopic follow-up. For evaluating the efficacy of endoscopy, primary hemostasis and recurrent bleeding were determined based on previous reports [8]. The following apparatus/materials were used for endotherapy: GIF-260J or GIF-260 (Olympus, Japan); Sclerotherapy Needle and Interject™ Injection Therapy Needle Catheter (Boston Scientific, USA); Resolution™ Clip Hemoclip (Boston Scientific, USA); and N-butyl-2-cyanoacrylate 0.5 mL (Braun, German).

### 2.4. Statistical Analysis

GraphPad Prism 5.01 software was used for the statistical analysis. For comparing the differences in qualitative data between the two groups, chi-squared test or Fisher's exact test was performed. Student's *t*-test was used for comparing the mean differences between the two groups. *P* < 0.05 was considered statistically significant.

## 3. Results

### 3.1. Demographic Data

A total of 18 duodenal DL patients from three medical centers met the inclusion criteria. Patient's demographic data are shown in Table 1. The major manifestation was melena (61.11%), hematochezia (22.22%), and hematemesis and melena (16.67%). Comorbidities of duodenal DL were observed in 12 patients (66.67%), including hypertension in 2 patients (11.11%), diabetes mellitus in 3 patients (16.67%), hepatic cirrhosis in 4 patients (22.22%), fat liver in 2 patients (11.11%), cholelithiasis in 1 patient (5.56%), arteriosclerosis in 3 patients (16.67%), and hyperlipoidemia in 2 patients (11.11%). The compensated shock was observed in three patients (16.67%). No significant difference was observed between the two groups with regard to age, sex, hemoglobin and PLT levels, and comorbidities (Table 1).

### 3.2. Endotherapy

**Figure 1:**
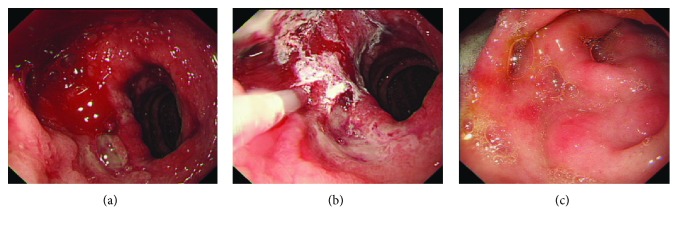
A male (58 years old) duodenal DL patient received cyanoacrylate injection during upper endoscopy. (a) An active bleeding was observed on the descending part of the duodenum. (b) Cyanoacrylate injection. (c) One month after endotherapy.

**Figure 2:**
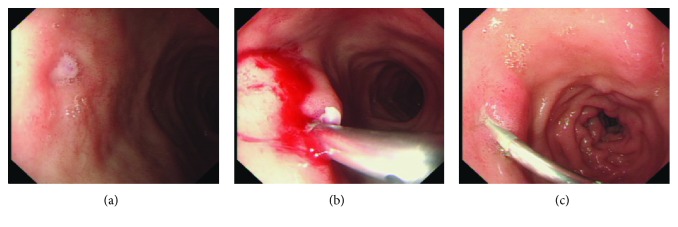
A male (28 years old) duodenal DL patient received hemoclip placement during upper endoscopy. (a) A protruding vessel 1 month post endoscopy, without active hemorrhage on the superior part of the duodenum. (b) Hemoclip placement. (c) 10 days after endotherapy.

All endotherapies at three medical centers were performed with standard upper endoscopes by experienced gastroenterologists. Based on the present data, the use of hemoclips or cyanoacrylate injection usually depends on the preference of endoscopists; two patients with active bleeding received cyanoacrylate injection in the CI group (Table 1, Figure 1). In the HP group, there were 12 patients (Figure 2), and totally, 16 hemoclips were used; 8 patients had active bleeding, 3 patients had a protruding vessel, and one patient had a clot (Table 1, Figure 2). In addition to 6 patients from the CI group, 4.5 mL (0.5 mL × 9) was used in total. None of the patients needed surgery or any other additional treatment. There were no marked side effects after our endoscopic treatment, except that one patient in the CI group developed mild ulcer at the injection site and completely recovered after rabeprazole therapy.

### 3.3. Hemostasis Rate and Rebleeding Rate

All patients received first endoscopic examination and/or endotherapy within 12 hours after being admitted into these medical centers. To observe the effects of CI and HP to duodenal DL, we compared primary hemostasis and recurrent hemorrhage between the two groups. As shown in Table 2, no differences were observed between the CI and the HP groups in primary hemostasis or recurrent hemorrhage, except for 1 patient from the HP group, who had melena reoccur three days after the first endotherapy; thus, cyanoacrylate injection was performed once again. The clinical outcome was summarized in Table 2.

## 4. Discussion

Before the 1980s, a surgical approach was the only definitive treatment for DL patients failed by drugs, and the effectiveness of angiographic embolization was usually disappointing [9]. During that time, a surgical operation was considered the only life-saving option [10]. In the late 1980s, endoscopic electrocoagulation was first used for controlling hemorrhage in DL patients, and a selective arterial embolization was the alternative treatment used for failed endoscopic therapy [11]. After that, other endoscopic therapies have been used in clinical DL treatment as well, including epinephrine injection, heater probe technique, bipolar electrocoagulation, and Nd : YAG laser photocoagulation [12]. During the late 1990s, clipping and ethanol injection or sclerotherapy was introduced into the treatment of DL bleeding [13, 14]. However, subsequent retrospective analysis has suggested that endoscopic thermal coagulation should be the first choice of initial treatment for DL hemorrhage [15]. Hemoclip [16, 17] and band ligation [18] have also gradually become commonly used therapies for DL treatment [19, 20].

Since most of DLs are located in the stomach [21, 22], there are almost no reports on the methods of duodenal DL treatment before the 1990s. In 1990, Goldenberg et al. have reported that a patient with duodenal DL was successfully treated with endoscopic injection of epinephrine and electrocoagulation [23]. Several years later, Hokama et al. have combined endoscopic clipping and ethanol injection in treating duodenal DL [24]. At the beginning of this century, endoscopic band ligation also became widely used for treating duodenal DL [25]. Nevertheless, further controlled studies are needed to evaluate the different methods or combined therapies. Recently, the first clinical controlled study about endotherapy of duodenal DL treatment has been published [26], showing that endoscopic band ligation and hemoclip placement are equally effective in duodenal DL treatment.

Cyanoacrylate injection has been used to treat hemorrhage of esophageal varices since the late 1970s [27]. This technique is a standard method to control varix bleeding in cirrhotic patients worldwide [28, 29]. However, there has been no clinical evaluation of its effectiveness in treating duodenal DL, except for a recent case report [30]. In the present study, we retrospectively analyzed the effectiveness of cyanoacrylate injection versus hemoclip placement in duodenal DL treatment. Based on our observation, there was no difference in effectiveness between the CI and the HP groups. In all of our cases, hemorrhage was completely controlled after cyanoacrylate injection. Moreover, no noticeable side effects were observed in either group. It seems that 0.5 mL cyanoacrylate (usual dosage per patient) was more expensive than one hemoclip.

The major limitation of the present retrospective study was patient grouping. Only six patients received cyanoacrylate injection, and eight patients with active bleeding received hemoclip placement. It seems that the patient grouping (with or without active bleeding) may lead to a selection bias. As a new method, cyanoacrylate injection was mainly used to control active hemorrhage, such as varix bleeding. More importantly, compared with other endoscopic hemostatic methods in a recent meta-analysis report, cyanoacrylate injection is associated with increased likelihood of hemostasis of active bleeding [31]. Moreover, according to a recent study, there is no difference in the hemostasis rate in acute nonvariceal upper gastrointestinal bleeding between cyanoacrylate injection and hemoclip placement [32]. In the present study, the data indicated that the patient grouping did not lead to significant clinical bias in the hemostasis rate.

In conclusion, both CI and HP are effective approaches for treating duodenal DL. CI has a higher hemostasis rate without significant side effects. Further large sample size and prospective randomized trials are necessary to evaluate the efficacy and safety of duodenal DL treatments.

## Figures and Tables

**Table 1:** Clinical characteristics of patients in the cyanoacrylate injection and hemoclip placement groups.

	CI (*n* = 6)	HP (*n* = 12)	*P* value
Age (years)	51.50 ± 7.29	44.58 ± 12.14	0.221
Blood pressure (mmHg)	109.8 ± 32.73	104.3 ± 14.37	0.616
Hemoglobin level (g/dL)	86.43 ± 30.15	88.30 ± 37.21	0.917
Platelet	177.0 ± 52.21	213.8 ± 104.9	0.434
Prothrombin time	13.93 ± 2.34	13.25 ± 2.45	0.579
Concurrent disease	4	8	1.00
Endoscopic characteristic			1.00
Active hemorrhage	2	8	
Protruding vessel without active hemorrhage	3	3	
Blood clot	1	1	
Location			0.344
Duodenum			
Duodenal bulb	3	9	
Descendant duodenum	3	3	

**Table 2:** Clinical outcomes of cyanoacrylate injection and hemoclip placement on Dieulafoy's lesions.

	CI (*n* = 6)	HP (*n* = 12)	*P* value
Primary hemostasis	6	11	1.00
Recurrent hemorrhage	0	1	
Transfusion (mL)	*N* = 2, total 4 IU	*N* = 1, total 1 IU	
Hospital stay (days)	9.833 ± 1.11	9.333 ± 1.45	0.824

## Abbreviations

DL: Dieulafoy's lesion.
